# A Dual Role of the Senescence Marker P16Ink4a in Liver Endothelial Cell Function

**DOI:** 10.3390/cells13231929

**Published:** 2024-11-21

**Authors:** Kay-Dietrich Wagner, Hasan Safwan-Zaiter, Nicole Wagner

**Affiliations:** CNRS, INSERM, iBV, Université Côte d’Azur, 06107 Nice, France; bc.hasansafwan@gmail.com

**Keywords:** p16Ink4a, endothelial cell, liver, senescence, proliferation, reactive oxygen species, endothelial cell leakage, loss-of-function, gain-of-function, migration, inflammation

## Abstract

P16Ink4a is a well-established marker of senescence. Although P16Ink4a is expressed in endothelial cells, little is known about its function in these cells. Using isolated liver endothelial cells with silencing or overexpression of P16Ink4a, we show here that dependent on P16Ink4a levels, different pathways and functions are affected. High levels of P16Ink4a reduce proliferation and induce senescence, while low levels have the opposite effects. Only high P16Ink4a expression reduces in vitro angiogenesis. Expression profiling reveals an inflammatory phenotype upon silencing of P16Ink4a, while P16Ink4a overexpression is associated with a profile associated with DNA damage, repair and senescence. Low levels of P16Ink4a induce reactive oxygen species (ROS) generation and increase endothelial cell leakage. Collectively, P16Ink4a represents an “antagonistic pleiotropy” gene, which is, on the one hand, required to prevent ROS generation and endothelial damage and, on the other hand, inhibits angiogenesis through induction of senescence at high levels.

## 1. Introduction

Senescence is a stress response characterized by an irreversible arrest of cellular proliferation [1]. It was first described in cultured primary fibroblasts in response to extensive periods of culture and passaging [2,3]. It is widely accepted that senescence contributes to aging and age-related disease. Thus, targeting senescent cells became a hot topic for therapeutic exploitation [4]. However, according to the evolutionary “antagonistic pleiotropy” theory of senescence and aging, certain genes could have evolved because of beneficial early properties in life, but they also might have deleterious later effects [5,6]. Typical established markers for aging and senescence are P16Ink4a and P21 [7,8,9,10]. They were both initially identified as cell cycle inhibitors [11,12]. P16Ink4a acts as a specific inhibitor of the cyclin-dependent kinases CDK4 and CDK6, subsequently inducing proliferation arrest by rendering retinoblastoma protein (pRB) in a hypo-phosphorylated state [13]. P21 acts as a downstream effector of P53 in cell cycle control and senescence [14]. In agreement with the antagonistic pleiotropy theory, the senescence marker P16Ink4a is frequently inactivated in human tumors [15], and *P16Ink4a* knockout mice die prematurely due to malignancies, mainly soft tissue tumors and angiosarcomas [16]. Besides P16Ink4a knockout mice, several mouse models have been developed to specifically ablate p16Ink4a expressing cells [17,18,19,20]. In these animals, several beneficial effects in aging have been described but also a reduction in health span, a requirement for P16Ink4a expressing cells in wound healing, and p16Ink4a-dependent promotion of epithelial stem cell regeneration in the lung (reviewed in [21]).

We recently investigated P16Ink4a expression systematically in different organs during embryonic and postnatal development in adult and aged mice [22]. In agreement with the literature, P16Ink4a was highest in old animals but also showed a dynamic expression pattern in development. In the adult and aging populations, P16Ink4a was consistently localized in endothelial cells in different organs with the highest expression in liver endothelial cells. Although it had been shown before that ablation of cells with high expression of P16Ink4a results in defects of the endothelial cell layer lining, the vessels in the liver and fibrosis [19], this model, which eliminated the endothelial cells, is not informative to clarify the functional relevance of P16Ink4a in endothelial cells. For this purpose, we established here primary cultures of liver endothelial cells with silencing or overexpression of P16Ink4a and show that, dependent on P16Ink4a expression levels, the endothelial cells show distinct functional characteristics.

## 2. Materials and Methods

### 2.1. Mouse Liver Endothelial Cell Isolation and Culture

All animal work was conducted according to national and international guidelines. C57BL/6 mice were purchased from Janvier Labs (Le Genest-Saint-Isle, France). Animals were euthanized by cervical dislocation and the livers dissected. Mouse liver endothelial cells (Cd31+) [22,23] and liver endothelial sinusoidal cells (Cd146+) [24] were isolated from livers of 3-month-old female mice. Liver samples were cut into approximately 1 mm^3^ fragments and digested with collagenase A and type I DNase (1 mg/mL and 100 IU/mL, respectively, Roche Diagnostics, Meylan, France) for 60 min at 37 °C. Cd31+ and Cd146+ cells were then purified from the cell suspension using Cd31 (Cat: 130-097-418) or Cd146 (Cat: 130-092-007) MicroBeads followed by magnetic separation in LS columns (Miltenyi Biotec SAS, Paris, France). Cd31+ and Cd146+ cells were grown in RPMI medium (Invitrogen, Cergy Pontoise, France) supplemented with 10% fetal calf serum (FCS), 100 IU mL^−1^ penicillin, and 100 µg mL^−1^ streptomycin (Invitrogen, Cergy Pontoise, France).

### 2.2. Lentivirus Production

Lentivirus for the transfection was prepared essentially as described [25]. An amount of 8.6 µg of pLenti-C-mGFP-P2A-Puro (cat.: PS100093) vector expressing “empty vector”, and P16 cDNA in the same vector backbone (cat.: MR201412L4, OriGene Technologies GmbH, Herford, Germany), or pLKO.1 vector (Sigma, St. Louis, MO, USA), expressing P16shRNA or non-coding shRNA, 8.6 µg of Lenti-Delta 8.91 and 2.8 µg of VSV-g (obtained from Didier Trono, Swiss Institute of Technology, Lausanne, Switzerland) were introduced into 5 × 10^6^ HEK 293 cells in DMEM, supplemented with 10% fetal calf serum at 37 °C in 5% CO_2_ in 10 cm dishes, through calcium phosphate-mediated transfection as described [25]. Virus-containing supernatants were collected after 48 h, passed through a 0.45 µm Millipore filter and used to infect Cd31+ and Cd146+ cells. The efficiency of infection was determined by Puromycin selection.

### 2.3. Migration Assays

One week after transduction, scratch migration assays [26] were performed. Images were captured after 0, 3, 6, 9, 12 and 24 h (Leica DMi8 microscope, with a Leica DFC7000T camera, and LASX software Version 1.4, Leica Microsystems, Wetzlar, Germany) and scratch diameters measured. Alternatively, transduced cells were seeded in the upper compartment of a modified Boyden chamber (5 × 10^4^ cells/chamber, pore size 8 µm, Corning Costar, SchipholRijk, The Netherlands). After 24 h, cells remaining in the upper chamber were scraped off, and the migrated cells at the bottom side fixed with PFA (Sigma), followed by staining with DAPI as described [27]. Quantification was performed by counting five random fields of nine independent experiments. 

### 2.4. In Vitro Angiogenesis Assays

Twenty-four hours after transduction, Cd31+ and/or Cd146+ cells were transferred to 96-well dishes coated with 100 µL Matrigel Basement membrane matrix (Cat: CLS354234) Chemicon, Millipore, Guyancourt, France). Branching points, tubule number, and tubule length were determined after additional 6 h as described [25,26]. Quantification was performed for five random fields of nine independent experiments for each condition using ImageJ (Version 1.53) with the Angiogenesis Analyzer Plugin [28].

### 2.5. SDS–Polyacrylamide Gel Electrophoresis and Western Blot

Total cell lysates were prepared, electrophoresed and blotted, as described in detail elsewhere [22]. The following antibodies were used: P16, rabbit monoclonal, (1:2000, Abcam), b-actin, rabbit polyclonal (1:500, Santa Cruz Biotechnology, Heidelberg, Germany), and tubulin, mouse monoclonal (1:2000, Sigma). Western blot bands were quantified relatively using ImageJ [29].

### 2.6. RNA Isolation, Reverse Transcription, and Quantitative Polymerase Chain Reaction (PCR)

Trizol reagent (Invitrogen) was used to isolate total RNA. cDNA synthesis was performed with 0.5 µg of total RNA (Thermo Scientific Maxima First Strand cDNA synthesis kit). An amount of 2.5 ng of cDNA was used for real-time PCR amplification (StepOne plus, Thermo Fisher Scientific, Illkirch, France) using SYBR^®^ Select Master Mix (Thermo Fisher Scientific, Illkirch, France. Expression of each gene was normalized to the respective arithmetic mean of Gapdh, Actnb, and Rplp0 expression [30]. Primer sequences are listed in Table 1.

### 2.7. Apoptosis Assays

One week after transduction, Cd31+ and Cd146+ cells were incubated with annexin V (APC-conjugated) and propidium iodide (Cat: 88-8007-72, Thermo Fisher Scientific, Illkirch, France) to mark apoptotic and necrotic cells and subsequently analyzed by flow cytometry as described [23].

### 2.8. Reactive Oxygen Species Measurement

Equally one week after transduction, Cd31+ and Cd146+ cells were incubated with CellROX^TM^ Deep Red Reagent (Cat: C10422, Thermo Fisher Scientific, Illkirch, France) for 30 min at 37 °C according to the manufacturer’s instructions. Cells were analyzed by flow cytometry (Fortessa, BD Biosciences, Le Pont de Claix, France). 

### 2.9. Detection of Cell Proliferation

After 1 week of transduction, bromodeoxyuridine was added and the cells incubated for 3 h. BrdU incorporation was measured spectrophotometrically according to manufacturer’s instructions (Cat: 11647229001, Millipore, Molsheim, France). Additionally, cell counts were performed using disposable cell counting slides and an inverted microscope (Axiovert, Zeiss, Jena, Germany) over a period of two weeks, starting after cell transduction.

### 2.10. Cellular Senescence Assays

Following one week of transduction, senescence was characterized by the appearance of senescence-associated β-galactosidase (SA-β-gal) activity using a Cellular Senescence Assay Kit according to the manufacturer’s instructions (Cat: KAA002, Sigma Aldrich, Molsheim, France).

### 2.11. Endothelial Cell Permeability Assays

Endothelial cell permeability of transduced cells was investigated using a Transwell permeability assay as described [31]. Briefly, cells were seeded into 6.5 mm Transwell^®^ inserts with 0.4 µm polycarbonate membranes in 24-well plates (Cat: 3413, Corning) and grown to confluence. Afterwards, streptavidin–horseradish peroxidase (HRP) (Cat: DY998, R&D Systems, Bio-Techne SAS, Noyal Châtillon sur Seiche, France) was added to the upper compartment. After 10 min, 20 µL aliquots were collected from the lower compartment, transferred into 96-well plates and 3,3′,5,5′-tetramethylbenzidine (TMB) substrate (Cat: T0440, Sigma-Aldrich, Molsheim, France) added. The color reaction was stopped by adding 25 µL of 2 N H_2_SO_4_ and the plate measured at 450 nm in an Elisa reader (iMark™, Biorad, Gémenos, France).

### 2.12. mRNA Sequencing

For sequencing, RNAs from liver sorted endothelial cells (Cd31+) and liver endothelial sinusoidal cells (Cd146+) 1 week after transduction were used (*n* = 6 each). RNA quality controls, sequencing, and data analysis was performed by Novogene (Cambridge, UK) as described [23]. 

### 2.13. Statistical Analysis

Data are expressed as mean ± SEM. Student’s *t*-tests (Instat 3.0, GraphPad) were used to determine statistical significance. A *p*-value of less than 0.05 was considered significant.

## 3. Results

**Figure 1:**
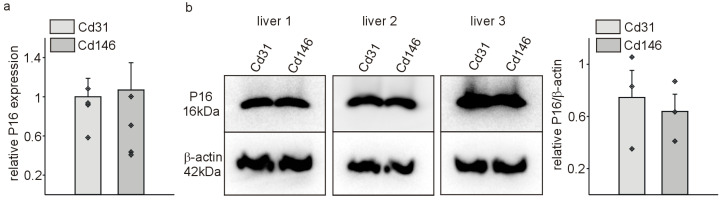
Liver vascular Cd31+ and Cd146+ cells express comparable levels of P16Ink4a. (**a**) Quantitative RT-PCRs for *p16Ink4a* of sorted liver endothelial Cd31+ and Cd146+ cells at 3 months of age of the mice. Expression of *p16Ink4a* was normalized to the respective means of *Gapdh*, *actin*, and *Rplp0* expression. Data are mean ± SEM (*n* = 4 each). Symbols indicate individual values. (**b**) Western blot for P16Ink4a in isolated Cd31+ and Cd146+ liver endothelial cell populations from 3-month-old mice. β-actin served as standard (left panel) and relative quantification of the Western blot bands (right panel graph). Data are mean ± SEM (*n* = 3 each). Symbols indicate individual values.

P16Ink4a expression has been described in liver endothelial cells, and elimination of the cells expressing high levels of P16Ink4a was associated with reduced health span of mice [19]. In this model, rare P16Ink4a-positive cells were detected in the liver at the age of 2 months [19], while we detected robust P16Ink4a expression in liver endothelial cells by immunohistochemistry and Western blot more recently at the age of 3 months [22]. Thus, we used the same age and gender of mice in the present study to investigate potential functions of P16Ink4a in liver endothelial cells. We isolated and cultured the main Cd31+ (general endothelial cell population) and Cd146+ (liver sinusoidal endothelial cells) from adult mice [23,24]. First, we determined whether the isolated cells corresponding to the in vivo situation [22] expressed P16Ink4a. Using quantitative RT-PCR, we detected comparable P16Ink4a mRNA levels in Cd31+ and Cd146+ isolated cells (Figure 1a). This result was confirmed in cells from different animals on the protein level by Western blot (Figure 1b). We detected robust P16Ink4a protein expression in both cell populations without significant differences between Cd31+ and Cd146+ cells.

**Figure 2:**
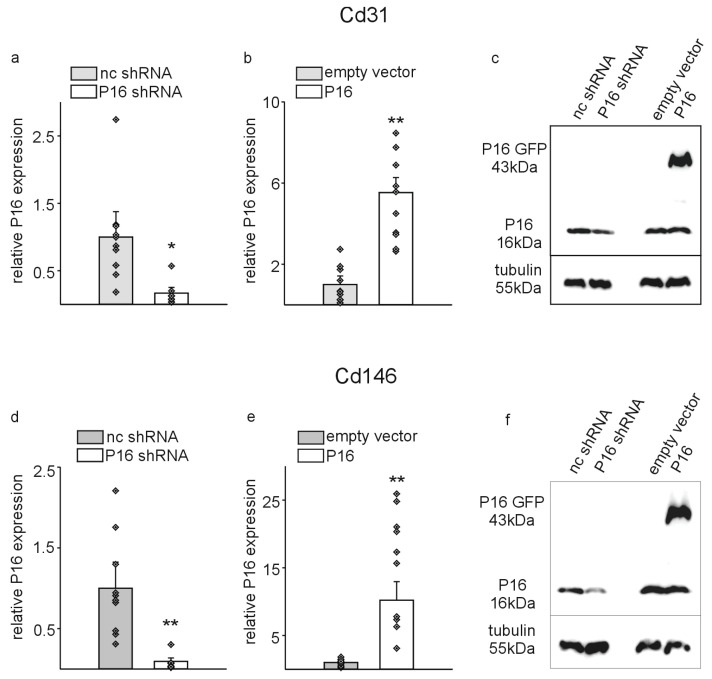
Lentiviral transduction with small hairpin RNA (shRNA) or P16Ink4a overexpression constructs (P16) efficiently modifies P16Ink4a expression levels in Cd31+ and Cd146+ liver endothelial cells. Quantitative RT-PCRs for P16Ink4a of Cd31- (**a**,**b**) and Cd146- (**d**,**e**) sorted liver endothelial cells. RNA was extracted one week after lentiviral transduction. Expression of P16Ink4a was normalized to the respective mean of Gapdh, actin, and Rplp0 expression. The average of the non-coding small hairpin construct (nc shRNA) transduced cells was calculated and set to 1. Individual samples of non-coding construct transduced or P16 small hairpin (P16 shRNA) transduced cells were then normalized against this average value (**a**,**d**). For the overexpression experiments, the average of the empty vector transduced cells was calculated and set to 1. All samples of empty vector transduced or P16 overexpression construct transduced cells were then normalized against this average value (**b**,**e**). Data are mean ± SEM (*n* = 10 each). * *p* < 0.05, ** *p* < 0.01. Symbols indicate individual values. Western blot for P16Ink4a in Cd31+ (**c**) and Cd146+ (**f**) liver endothelial cells transduced with a P16 small hairpin construct (P16 shRNA) and the respective non-coding control (nc shRNA) or with a P16Ink4a overexpression construct (P16) and the corresponding empty vector control. Tubulin served as standard. Note that the P16 overexpression construct is a fusion between the P16Ink4a cDNA and green fluorescent protein (GFP) resulting in a band at the expected size of 43 kDa.

To modify P16Ink4a expression levels in the different endothelial cell populations as a prerequisite to investigate the function of this protein in these cells, we transduced the cells with lentiviral particles encoding for P16Ink4a small hairpin constructs (p16 shRNA) compared to non-coding particles (nc shRNA) or p16-GFP fusion constructs for overexpression (p16). In this case, the identical empty overexpression vector without the P16Ink4a cDNA insert served as control (empty vector). The most significant inhibition and overexpression were observed in both cell types seven days after transduction (Figure 2). Silencing by shRNA constructs reduced P16Ink4a expression on average by 80% in Cd31+ and Cd146+ cell populations compared to cells transduced with the non-coding constructs (Figure 2a,d). Transduction with the p16-GFP construct resulted in a significant overexpression of P16Ink4a in both cell populations on the RNA level (Figure 2b,e). Again, the results were confirmed on the protein level by Western blot experiments (Figure 2c,f). Of note, the overexpression did not affect the bands of the endogenous P16Ink4a protein, which was detectable at 16 kDa, but a significant additional band was evident at 43 kDa for the p16-GFP fusion protein compared to the empty vector controls.

**Figure 3:**
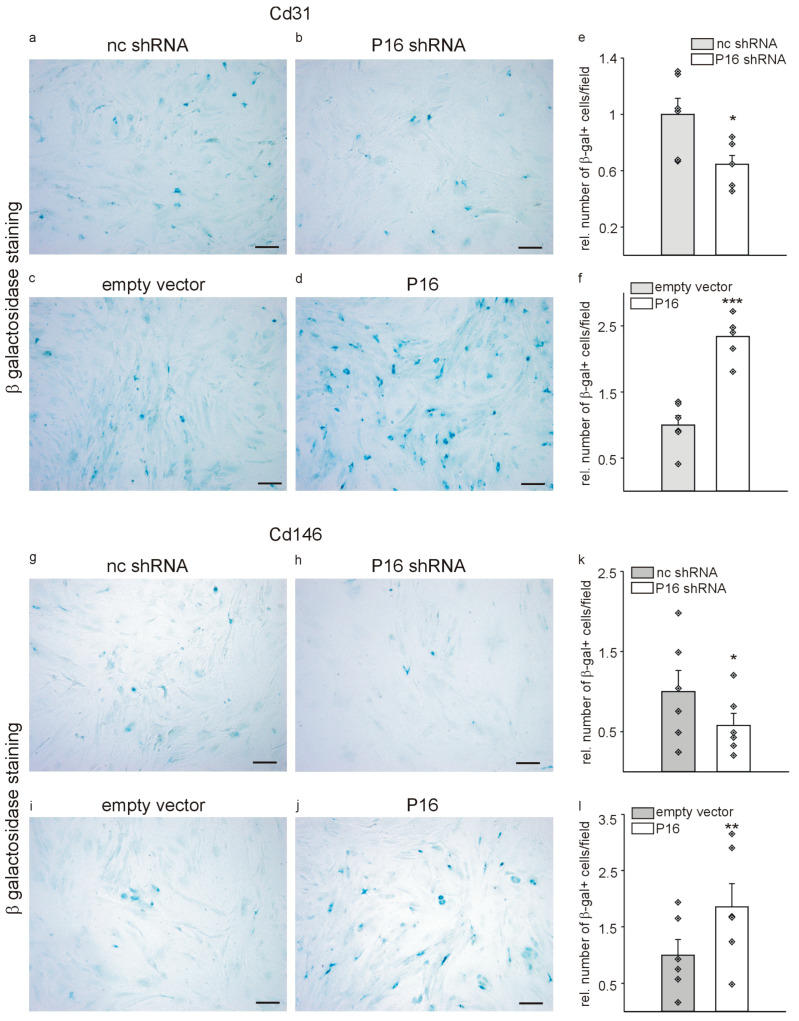
SA-β-galactosidase staining of Cd31+ and Cd146+ liver endothelial cell populations with modified P16Ink4a expression as marker for senescence. Cd31+ cells were transduced with lentiviral particles containing non-coding small hairpin constructs (nc shRNA, (**a**)), P16Ink4a silencing plasmids (P16 shRNA, (**b**)), empty vector controls (**c**) for the P16Ink4a overexpression construct (P16, (**d**)). Quantification of the number of SA-β-galactosidase positive Cd31+ cells with silencing of P16Ink4a compared to the non-coding control (**e**) and overexpression of P16Ink4a compared to the corresponding empty vector control (**f**). Representative SA-β-galactosidase staining of Cd146+ cells transduced with non-coding control constructs (**g**), P16Ink4a small hairpin silencing plasmids (**h**), empty vector controls (**i**) corresponding to the P16Ink4a overexpression construct (**j**). Quantification of the number of SA-β-galactosidase positive Cd146+ cells with silencing of P16Ink4a compared to the non-coding control (**k**) and overexpression of P16Ink4a compared to the corresponding empty vector control (**l**). Scale bars represent 50 µm. Data are mean ± SEM (*n* = 6, each). Symbols indicate individual values. * *p* < 0.05; ** *p* ˂ 0.01; *** *p* ˂ 0.001.

As P16Ink4a is commonly considered as a marker for senescence [17,32,33,34,35,36,37,38], we performed SA-β-galactosidase staining of Cd31+ and Cd146+ cell populations transduced with either p16 small hairpin or overexpression constructs and the respective controls. As reported in the literature [7], the overall fractions of SA-β-galactosidase-positive cells were low under control conditions for both cell types (approximately 1%). Interestingly, silencing of P16Ink4a further reduced the fraction of SA-β-galactosidase positive Cd31+ and Cd146+ cells compared to the respective control cells (Figure 3a–c,g–i). Overexpression of P16Ink4a significantly increased the fraction of SA-β-galactosidase-positive Cd31+ cells (Figure 3d–f) as well as of Cd146+ cells (Figure 3j–l), which is comparable to the situation in vivo in aged animals [19].

**Figure 4:**
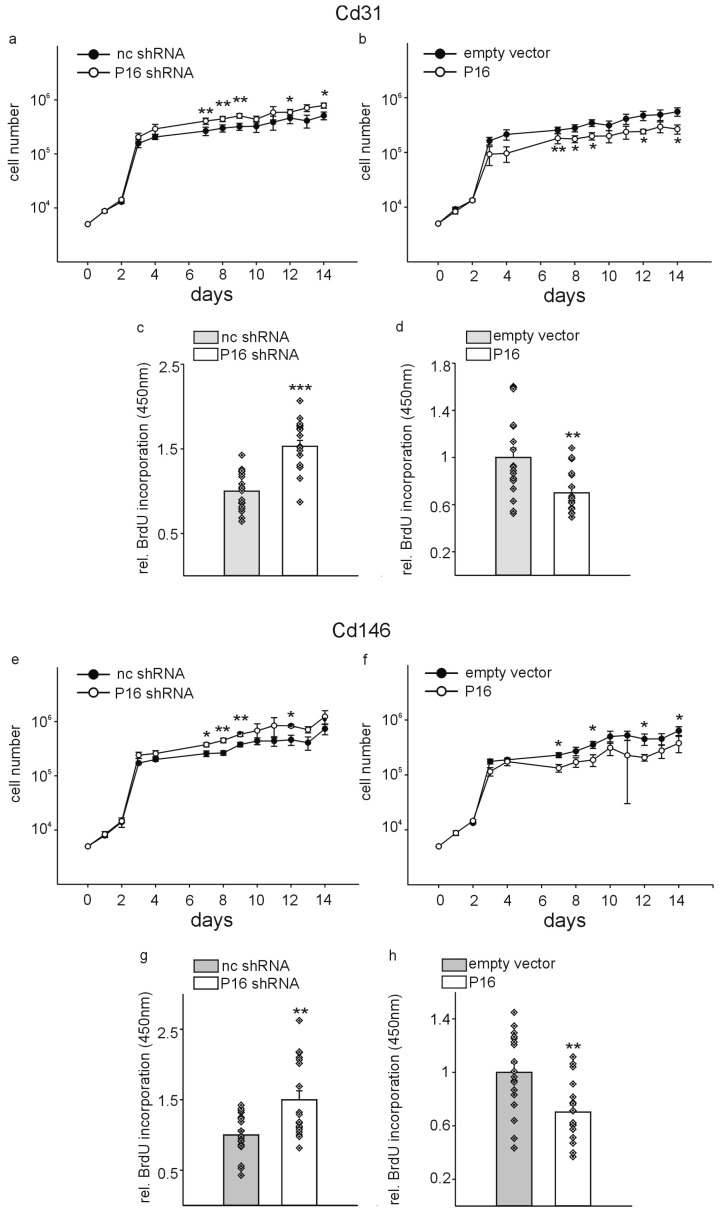
Modification of P16Ink4a levels alters proliferation of liver endothelial cells. Cd31+ were transduced with lentiviral particles containing non-coding small hairpin constructs (nc shRNA) or P16Ink4a silencing plasmids (P16 shRNA) (**a**), or empty vector controls (empty vector) and a P16Ink4a overexpression construct (P16) (**b**). A total of 5 × 10^3^ cells were seeded in 96-well plates and the cells counted daily for two weeks to establish the cell growth curves. (*n* = 12, each). BrdU incorporation was measured after one week of the cell transduction as independent measure for cell proliferation in Cd31+ cells with silencing of P16Ink4a compared to the respective control (**c**) and in cells with P16Ink4a overexpression compared to the corresponding empty vector controls (**d**) (*n* = 18, each). Growth curves of Cd146+ cells with silencing (**e**) or overexpression (**f**) of P16Ink4a (*n* = 12, each). Cells were subjected to the same experimental conditions as for the Cd31+ cells. BrdU incorporation in Cd146+ cells with silencing of P16Ink4a compared to the respective control (**g**) and in cells with P16Ink4a overexpression compared to the corresponding empty vector controls (**h**) (*n* = 18, each). Data are mean ± SEM. Symbols indicate individual values. * *p* < 0.05; ** *p* ˂ 0.01; *** *p* ˂ 0.001.

Besides the well-known role of P16Ink4a as a senescence marker, the protein was originally identified as a tumor suppressor and regulator of the cell cycle [13] (reviewed in [10]). Thus, we reasoned that modulation of P16Ink4a expression might affect the cell growth of the different endothelial cell populations. To investigate this possibility, we used a comparable approach as before and transduced multiple wells of 96-well plates containing 5 × 10^3^ Cd31+ or Cd146+ cells with lentiviral particles containing p16 small hairpin or overexpression constructs and the respective controls. Afterwards, cells were counted daily for 2 weeks (Figure 4a,b,e,f). Initially, before the silencing or overexpression became effective, we did not observe significant differences in cell numbers between the different groups. Beginning at one week after transduction, we observed significant higher cell numbers for Cd31+ and Cd146+ cells transduced with the P16Ink4a silencing construct compared to the respective controls, while P16Ink4a overexpression reduced the numbers in both cell types. Afterwards, these differences remained stable until the end of the two weeks’ observation period. To further confirm more directly the potential differences in cell proliferation due to P16Ink4a silencing or overexpression, we performed BrdU incorporation assays after one week of the transduction of both cell types. Silencing of P16Ink4a increased proliferation Cd31+ as well as Cd146+ cells compared to controls, while overexpression of P16Ink4a reduced the proliferation of both endothelial cell populations (Figure 4c,d,g,h).

**Figure 5:**
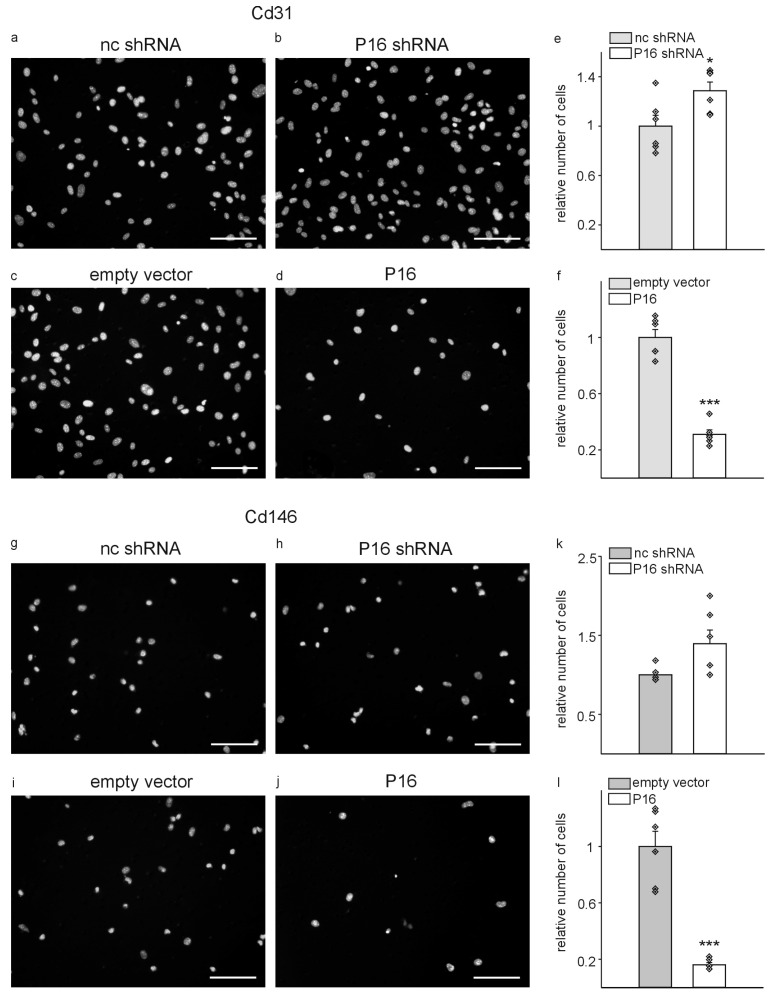
Migration of liver endothelial cells is affected by P16Ink4a. Representative photomicrographs of DAPI-stained Cd31 cells migrated through Transwell membranes. Cells transduced with non-coding small hairpin plasmids (nc shRNA) served as control (**a**) for P16Ink4a silencing plasmid (P16 shRNA) transduced cells (**b**). Empty vector controls (**c**) were compared to cells with overexpression of P16Ink4a (P16) (**d**). Quantification of the number of migrated cells with silencing of P16Ink4a (**e**) and with overexpression of P16Ink4a (**f**) compared to the respective controls. The same experimental approach was repeated with Cd146+ cells including nc shRNA (**g**), P16 shRNA (**h**), empty vector controls (**i**), and overexpression of P16Ink4a (**j**). Quantification of the number of migrated cells was repeated for Cd146+ cells with silencing (**k**) or overexpression of P16Ink4a (**l**) compared to the respective controls. Scale bars represent 50 µm. (*n* = 6, each). Data are mean ± SEM. Symbols indicate individual values. * *p* < 0.05; *** *p* ˂ 0.001. Note that although Cd146+ cells migrate less compared to CD31+ cells, P16Ink4a overexpression significantly inhibits endothelial cell passage through the Transwell membranes in both cell populations.

As endothelial cell migration is a fundamental mechanism of angiogenesis, vasculogenesis, and vessel repair [39] and removal of endothelial cells with high P16Ink4a expression causes vascular damage in the liver in vivo [19], we next investigated whether modulation of P16Ink4a expression might alter the migratory properties of the two liver endothelial cell populations. Silencing of P16Ink4a significantly enhanced the migration, while overexpression significantly diminished migration of Cd31+ cells compared to the respective controls in Transwell migration assays (Figure 5a–f). Cd146+ migrated less compared to Cd31+ cells. Nevertheless, silencing did not significantly increase the migration while overexpression of P16Ink4a significantly reduced the migration of Cd146+ cells (Figure 5g–l). 

**Figure 6:**
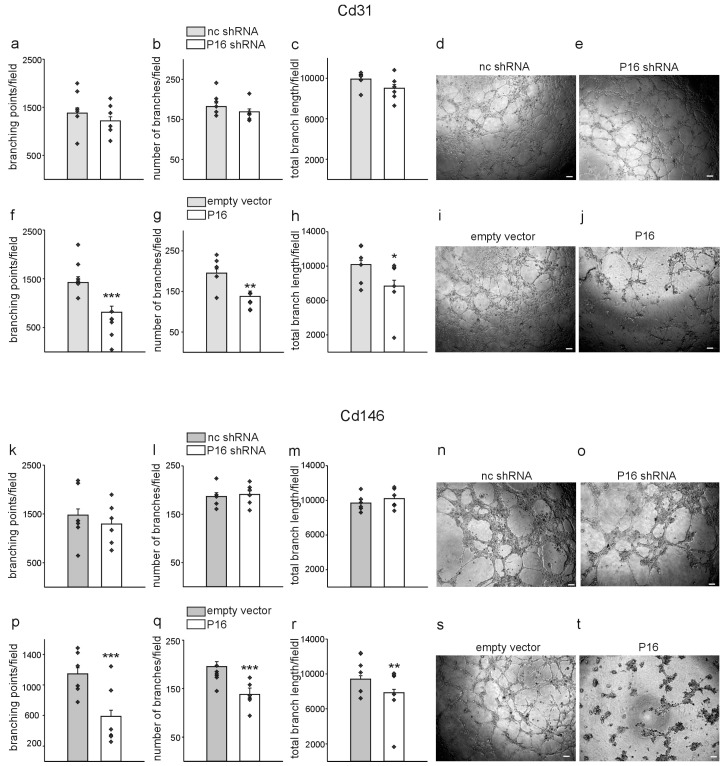
High P16Ink4a reduces in vitro angiogenesis of Cd31+ and Cd146+ liver endothelial cells. Matrigel tube formation assays as in vitro measure for angiogenesis. Branching points (**a**), number of branches (**b**), and total branch length (**c**) were quantified in Cd31+ cells with silencing of P16Ink4a (p16 shRNA) in comparison to the respective controls (nc shRNA). Representative photomicrographs of control endothelial tubes (**d**) and endothelial cells with silencing of P16Ink4a (**e**). Quantification of branching points (**f**), number of branches (**g**), and total branch length (**h**) of endothelial tubes formed from Cd31+ cells with overexpression of P16Ink4a (P16) and the respective controls (empty vector). Photomicrographs of empty vector transduced control Cd31+ cells (**i**) and cells with overexpression of P16Ink4a (**j**). Quantification of branching points (**k**), number of branches (**l**), and total branch length (**m**) of endothelial tubes formed from Cd146+ cells with silencing of P16Ink4a (p16 shRNA) in comparison to the respective controls (nc shRNA). Photomicrographs of Cd146+ control endothelial tubes (**n**) and endothelial cells with silencing of P16Ink4a (**o**). Quantification of branching points (**p**), number of branches (**q**), and total branch length (**r**) of endothelial tubes formed from Cd146+ cells’ overexpression of P16Ink4a (P16) and the respective controls (empty vector). Photomicrographs of empty vector transduced control Cd146+ cells (**s**) and cells with overexpression of P16Ink4a (**t**). Scale bars represent 50 µm. (*n* = 6, each). Data are mean ± SEM. Symbols indicate individual values. * *p* < 0.05; ** *p* < 0.01; *** *p* ˂ 0.001.

To further characterize the migratory properties of the two liver endothelial cell populations in response to modulation of the P16Ink4a expression levels, we performed wound healing (scratch) assays of confluent cell monolayers and measured the wound closure over time [40]. Representative pictures for the wound healing of Cd31+ cells are shown in Supplementary Figure S1a and for Cd146+ cells in Supplementary Figure S2a. The gap size was measured and quantified for all experimental conditions and the two cell types at 0, 3, 6, 9, 12, and 24 h after scratching of the monolayers. Quantification of the scar length revealed no significant differences for Cd31+ and Cd146+ cells with silencing of P16Ink4a compared to the respective non-coding controls (Supplementary Figures S1b and S2b), while overexpression of P16Ink4a delayed the wound closure in Cd31+ and Cd146+ cells (Supplementary Figures S1c and S2c). Next, we performed endothelial tube formation assays on basement membrane extracts, which represents the most commonly used in vitro angiogenesis assay [41]. For this purpose, Cd31+ and Cd146+ cells were plated one week after lentiviral transduction on Matrigel^®^ basement membrane coated wells, pictures taken after 9 h using phase-contrast illumination, and parameters of the developing endothelial tubes quantified. Silencing of P16Ink4a did not significantly affect the number of branching points, number of branches, and total branch length in Cd31+ cells (Figure 6a–e) nor in Cd146+ cells (Figure 6k–o) compared to the respective controls. In contrast, overexpression of P16Ink4a significantly reduced the number of branching points, the number of branches and the total branch length compared to the respective controls in Cd31+ (Figure 6f–j) as well as in Cd146+ cells (Figure 6p–t).

Given that P16Ink4a has also been linked to apoptosis in various cell lines [42,43,44], we sought to explore whether modulating P16Ink4a expression in the two liver endothelial cell populations might influence programmed cell death. For this purpose, Cd31+ and Cd146+ cells were incubated with annexin-V to detect apoptosis and propidium iodide to distinguish from necrosis. The cells were examined by flow cytometry. In accordance with the preceding experiments, the cells were analyzed one week following lentiviral transduction with P16Ink4a small hairpin or overexpression constructs. In both Cd31+ and Cd146+ cells, silencing of P16Ink4a exhibited a tendency to reduce apoptosis, while overexpression demonstrated the opposite effects, though these did not reach statistical significance (Supplementary Figure S3).

**Figure 7:**
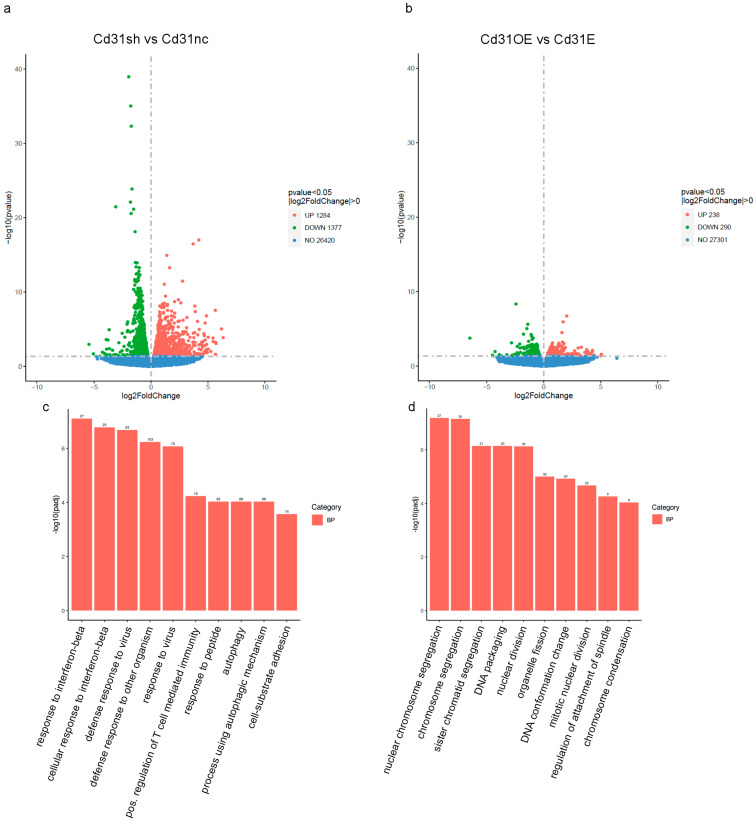

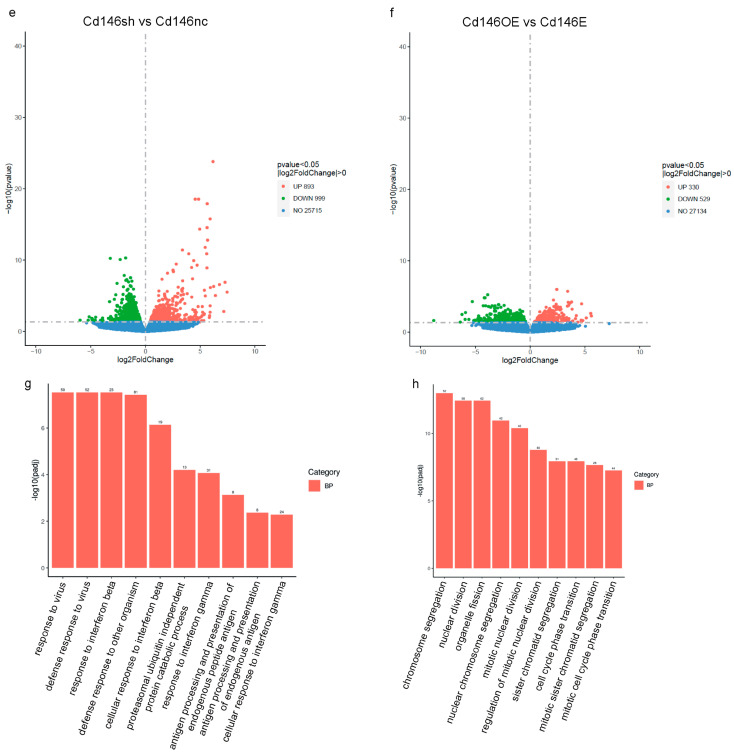
RNA sequencing analysis of Cd31+ (**a**–**d**) and Cd146+ (**e**–**h**) cells with modification of P16Ink4a expression levels (*n* = 3 each) corroborates the dual function of P16Ink4a in endothelial cells. Cells were transduced with lentiviral particles containing either P16 small hairpin (P16 shRNA) or overexpression constructs (P16) and the respective controls (nc shRNA, empty vector). (**a**) Volcano plot analysis of differentially expressed genes in Cd31+ cells with silencing of P16Ink4a (Cd31sh) compared to the respective controls (Cd31nc). (**b**) Volcano plot of differentially expressed genes in Cd31+ cells with overexpression of P16Ink4a (Cd31OE) and the respective control (Cd31E). (**c**) Cluster analysis of differentially expressed genes with the most significantly changed gene ontology terms (BP: biological process) in Cd31+ cells with silencing of P16Ink4a compared to the respective control and (**d**) cluster analysis of differentially expressed genes in Cd31+ cells with overexpression of P16Ink4a compared to the respective control. (**e**) Volcano plot of differentially expressed genes in Cd146+ cells with silencing of P16Ink4a (Cd146sh) compared to the respective controls (Cd146nc). (**f**) Volcano plot of differentially expressed genes in Cd146+ cells with overexpression of P16Ink4a (Cd146OE) and the respective control (Cd146E). (**g**) Cluster analysis of differentially expressed genes with the most significantly changed gene ontology terms (BP: biological process) in Cd146+ cells with silencing of P16Ink4a compared to the respective control and (**h**) cluster analysis of differentially expressed genes in Cd146+ cells with overexpression of P16Ink4a compared to the respective control.

To analyze the transcriptome of gene expression patterns in Cd31+ and Cd146+ cells in response to modulation of P16Ink4a levels, we isolated and cultured both cell types and transduced them with lentiviral constructs containing P16Ink4a small hairpin or overexpression constructs or the respective controls. mRNAs were submitted to sequencing after purity and quality controls. Silencing of P16Ink4a resulted in significant upregulation of 1284 genes and downregulation of 1377 genes (Figure 7a). Overexpression of P16Ink4a in Cd31+ cells induced upregulation of 238 genes and downregulation of 290 genes (Figure 7b). In Cd146+ cells, silencing of P16Ink4a caused upregulation of 893 and downregulation of 999 genes (Figure 7e), while overexpression of P16Ink4a provoked upregulation of 330 and downregulation of 529 genes in this cell population (Figure 7f). In both cell types, P16Ink4a silencing had a bigger impact on the number of affected genes compared to the overexpression. Interestingly, gene ontology term analysis of the involved biological processes revealed for both cell types that loss of P16Ink4a was associated with immunological processes in the endothelial cells (e.g., defence response to virus, response to interferon beta, antigen processing and presentation, etc.) (Figure 7c,g). In contrast, gene ontology term analysis of the biological processes affected by P16Ink4a overexpression was more in agreement with its known functions as cell cycle regulator and senescence mediator (e.g., chromosome segregation, nuclear division, cell cycle phase transition, etc.) (Figure 7d,h). In both endothelial cell types, silencing of P16Ink4a affected comparable biological processes (mainly related to immune function) as was also the case for P16Ink4a overexpression in both cell populations. Nevertheless, reduction in P16Ink4a levels or overexpression affected profoundly different biological processes, suggesting dual functions of P16Ink4a in endothelial cells dependent on the expression levels.

**Figure 8:**
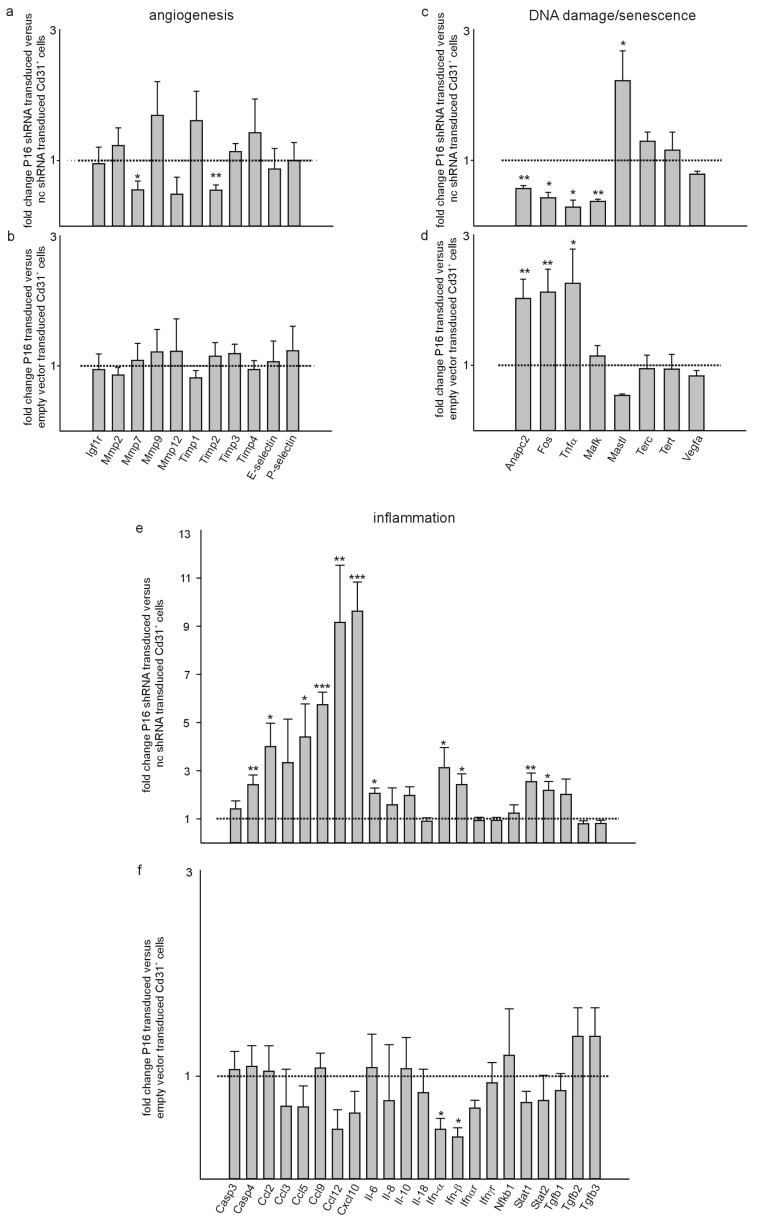

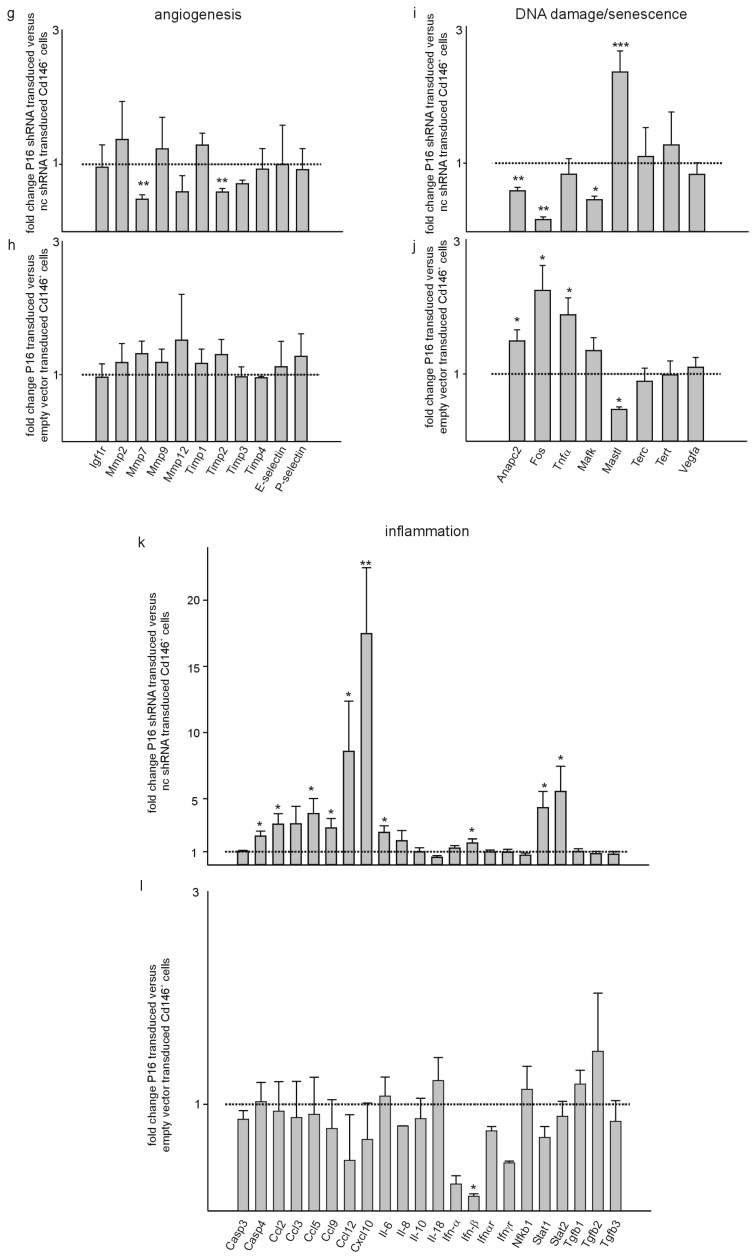
Quantitative RT-PCR corroborates inflammatory signatures upon silencing and DNA damage/senescence features upon overexpression of P16Ink4a. Quantitative RT-PCR of angiogenesis, DNA damage/senescence and inflammation markers of Cd31+ (**a**–**f**) and Cd146+ (**g**–**l**) liver endothelial cells with silencing or overexpression of P16Ink4a (*n* = 8 each). Data are mean ± SEM. ^∗^ *p* < 0.05, ^∗∗^ *p* < 0.01, ^∗∗∗^ *p* < 0.001.

Next, we verified some of the differential expressed genes by quantitative RT-PCR with a focus on factors involved in angiogenesis, DNA damage/senescence/cell cycle regulation and inflammatory responses based on our obtained results and the data from gene ontology clustering of the potentially involved biological processes. Of the investigated genes with a known function in angiogenesis, we observed a significant downregulation of Mmp7 [45] and Timp2 [46] in Cd31+ as well as Cd146+ cells with silencing of P16Ink4a compared to the respective controls (Figure 8a,g). Overexpression of P16Ink4a did not reveal significant differences for the investigated genes compared to empty vector transduced control cells (Figure 8b,h). TNF alpha expression was lower in Cd31+ cells but unchanged in Cd146+ cells silenced for P16Ink4a. Overexpression of P16Ink4a induced TNF alpha expression in both cell types, which is in agreement with a pro-senescent function of this molecule [47] (Figure 8c,d,i,j). Expression of the Mafk transcription factor was downregulated in both cell types upon silencing of P16Ink4a but not significantly affected by the overexpression of P16Ink4a. In contrast, in both cell types, inhibition of P16Ink4a expression induced expression of the Mastl kinase and P16Ink4a overexpression reduced expression of this kinase, which is implicated in cell cycle progression [48]. In agreement with our SA-β-galactosidase staining results, Anapc2 (anaphase promoting complex subunit 2) and Fos (FBJ murine osteosarcoma viral oncogene homolog) showed lower expression in both cell types with silencing of P16Ink4a, while overexpression had the opposite effects. Both factors are part of the senescence-associated secretory phenotype [49], which supports the notion that P16Ink4a overexpression induces senescence, but surprisingly, silencing of P16Ink4a can reduce senescence below the control level. Although Vegfa has been implicated in the vascular phenotype upon ablation of cells with high expression of p16 [19], we did not observe any alterations in Vegfa expression under the different conditions in both liver endothelial cell populations. Of note, several genes related to inflammatory responses were upregulated in both cell populations upon silencing of P16Ink4a, i.e., Casp4, Ccl2, Ccl5, Ccl9, Ccl12, Cxcl10, Il-6, Ifn-α, Ifn-β, Stat1, and Stat2. Overexpression of P16Ink4a, however, did not affect expression of any of the investigated genes related to inflammation in both cell populations (Figure 8e,f,k,l). 

**Figure 9:**
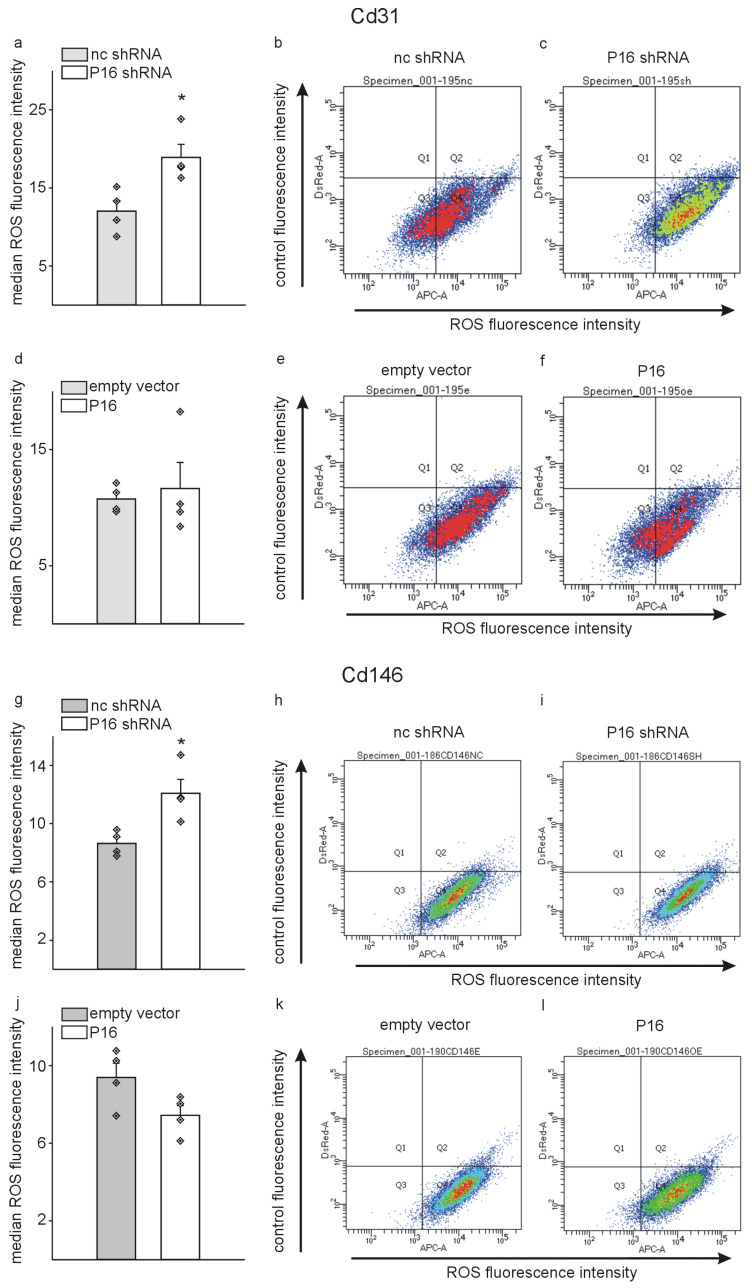
Reduction in P16Ink4a levels generates a higher amount of reactive oxygen species (ROS) in liver endothelial cells. Cd31+ (**a**–**f**) and Cd146+ (**g**–**l**) cells were incubated with CellROX^TM^ Deep Red reagent one week after transduction with P16Ink4a silencing or overexpression constructs and the respective controls and the signal was analyzed by flow cytometry. (**a**) Median values of ROS fluorescence in Cd31+ samples following silencing of P16Ink4a (P16 shRNA) and the respective control (nc shRNA). Representative examples for control (**b**) and P16Ink4a silencing (**c**). (**d**) Median values of ROS fluorescence in Cd31+ samples following overexpression of P16Ink4a (P16) and the respective control (empty vector). Representative examples for control (**e**) and P16Ink4a overexpression (**f**). (**g**) Median values of ROS fluorescence in Cd146+ samples following silencing of P16Ink4a and the respective control. Representative examples for control (**h**) and P16Ink4a silencing (**i**) in Cd146+ cells. (**j**) Median values of ROS fluorescence in Cd146+ samples following overexpression of P16Ink4a (P16) and the respective control (empty vector). Representative examples for control (**k**) and P16Ink4a overexpression (**l**). The data are presented as the mean ± SEM (*n* = 4, each). Symbols indicate individual values. * *p* < 0.05. Note that silencing of P16Ink4a increases ROS production as sign of endothelial damage in both cell populations.

As the mRNA sequencing and quantitative RT-PCR data suggest mostly a pro-inflammatory phenotype of endothelial cells in response to removal of P16Ink4a, we next investigated the generation of reactive oxygen species (ROS) in this context of a potential endothelial cell dysfunction [50]. For this purpose, after transduction, Cd31+ or Cd146+ cells were, as described above, incubated with CellROX^TM^ dye and the cells analyzed by flow cytometry. In agreement with the quantitative RT-PCR data, we measured a higher content of reactive oxygen species in Cd31+ and Cd146+ cells silenced for P16Ink4a, while overexpression of P16Ink4a did not significantly affect the ROS production compared to control in the two endothelial cell populations (Figure 9).

**Figure 10:**
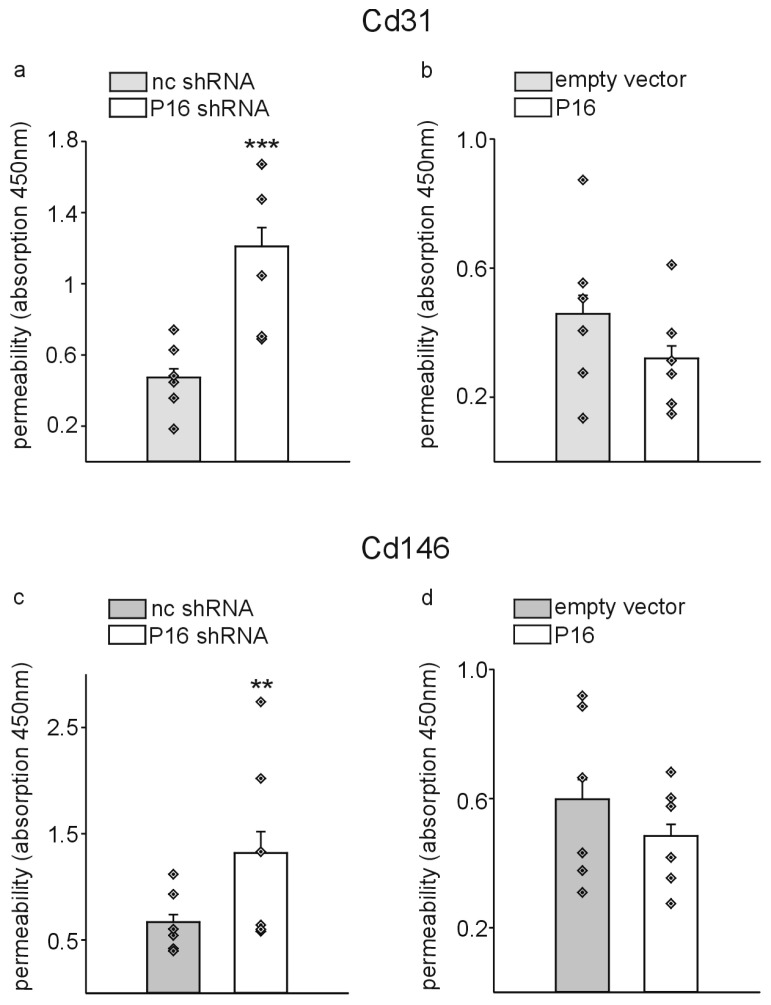
Reduction in P16Ink4a levels causes leakage in liver endothelial cells. Permeability of confluent CD31+ or Cd146+ liver endothelial cells was measured as streptavidin–horseradish peroxidase leakage in Transwell chambers following transduction with P16Ink4a silencing (P16 shRNA) (**a**,**c**) or overexpression constructs (**b**,**d**) and the respective controls (nc shRNA, empty vector). The data are presented as the mean ± SEM (*n* = 6, each). Symbols indicate individual values. ** *p* < 0.01, *** *p* < 0.001. Note that silencing of P16Ink4a significantly increased the permeability while overexpression had no significant effect.

Finally, to test whether the observed inflammatory gene expression profile and the increased ROS production upon silencing of P16Ink4a expression might have functional consequences for the endothelial cell populations, we employed Transwell permeability assays as described [31]. Basically, Cd31+ or Cd146+ cells were seeded on 0.4 µm polycarbonate membranes in 24-well plates and the cells grown until they formed a completely confluent monolayer. Afterwards, Streptavidin–horseradish peroxidase was added to the upper compartment of the chamber, and after 5 min, medium samples were collected from the lower compartment of the chamber, incubated with 3,3′,5,5′-tetramethylbenzidine and the absorbance measured spectrophotometrically to estimate the permeability of the cell monolayers. Silencing of P16Ink4a increased the permeability (leakage of streptavidin) significantly in Cd31+ as well as Cd146+ cells compared to the respective controls (Figure 10a,c). In contrast, overexpression of P16Ink4a had no significant effects on the endothelial cell permeability in both cell populations (Figure 10b,d).

## 4. Discussion

In accordance with our previous description, which indicated that the main cells expressing P16Ink4a in vivo are endothelial cells in mice of increasing age [22], we measured robust P16Ink4a mRNA and protein levels in isolated Cd31+ and Cd146+ liver endothelial cell populations in vitro. Given that both cell types express comparable levels of P16Ink4a and that silencing or overexpression was equally efficacious in both cell types, it is unsurprising that the biological responses to P16Ink4a modulation were analogous in terms of proliferation, senescence, angiogenesis, molecular markers, and endothelial cell damage.

In accordance with the initial identification of P16Ink4a as a cell cycle regulator and tumor suppressor [12,13,51], we observed a reduction in cell count upon overexpression and an increase in cell count upon silencing of P16Ink4a. It is noteworthy that these differences became evident and statistically significant one week after lentiviral transduction, which corresponds to the maximum silencing and overexpression effects for P16Ink4a, which were also detected at the same time point. The differences in cell growth and proliferation were confirmed at the same time point by measuring BrdU incorporation, which provides a direct measure of cell proliferation. The growth curves demonstrated that at the outset, both the CD31+ and CD146+ cell populations exhibited near-exponential growth. These cell populations were seeded at a very low density and transduced simultaneously. Subsequently, the growth curves reached a plateau, which is likely attributable to contact inhibition in response to increasing confluency [52]. Nevertheless, modulation of P16Ink4a expression resulted in effects that were fully consistent with the known function of this protein as a cell cycle regulator.

Furthermore, our data regarding the process of senescence, as revealed by SA-β-galactosidase staining in response to P16Ink4a modulation, are fully in agreement with the published literature (reviewed in [10,21]). The elevated expression of P16Ink4a was found to be correlated with an increased incidence of senescence. It is noteworthy that the silencing of P16Ink4a resulted in a reduction in the number of senescent cells to levels below those observed in the control group. This finding is in accordance with the established concept that the incidence of senescence increases gradually with age [4]. In vivo, a few P16Ink4a-expressing cells were detectable in mice at the age of two months, and dermal fibroblasts in culture exhibited increased P16Ink4a levels already at the second passage [19]. Although various P16Ink4a reporter systems in vivo exhibit considerable differences in sensitivity [21], our observations regarding proliferation and senescence in endothelial cells in culture are in agreement with the in vivo data, thereby validating our cell culture system for the investigation of P16Ink4a function in endothelial cells.

Although endothelial cells are predominantly quiescent in vivo, they retain the capacity to proliferate and are the primary cell type responsible for ensuring developmental and reparative angiogenesis. In vitro, proliferation, migration, and three-dimensional angiogenesis models are frequently employed to investigate the involvement of different molecules in angiogenic processes [53]. In order to investigate the potential contribution of endothelial P16Ink4a in angiogenesis, a combination of Transwell migration, scratch wound healing, and Matrigel tube formation assays was employed. While silencing of P16Ink4a had a limited impact on Transwell migration of the cells, the most notable differences were observed in the context of P16Ink4a overexpression. The forced expression of P16Ink4a resulted in the inhibition of Transwell migration, wound closure in the scratch wound healing assay, and in vitro angiogenesis in Matrigel angiogenesis assays. It can be postulated that the known increase in P16Ink4a in older individuals may be causative for the impaired angiogenesis and related pathologies observed in the aging process [54,55]. Further studies will hopefully elucidate this interesting possibility, which is highly relevant for potential clinical implications of senotherapies for cardiovascular disease (reviewed in [56]).

To gain a more comprehensive understanding of the global impact of P16Ink4a modulation on gene expression, which could have functional implications, we conducted mRNA sequencing of both endothelial cell populations with silenced or overexpressed P16Ink4a, compared to the respective controls. This was followed by gene ontology clustering of the potentially involved biological processes and validation of the expression differences by quantitative RT-PCR. In accordance with the known function of P16Ink4a as cell cycle regulator and marker of senescence, we observed consistent differences in genes related to cell cycle and SASP markers (Anapc2, Fos) upon overexpression of P16Ink4a compared to the respective controls. In line with our SA-β-galactosidase staining results, Anapc2 and Fos expression were not only higher in cells with P16Ink4a overexpression but reduced in cells with silencing of P16Ink4a, indicating that senescence is, at least in vitro, “titratable” dependent on P16Ink4a expression levels. Surprisingly, silencing of P16Ink4a resulted consistently, in both endothelial cell populations, in the upregulation of several inflammatory markers (Ccl’s, Cxcl10, Il-6) including caspase 4 [57]. Given the established link between caspase 4 and inflammatory marker expression and reactive oxygen species (ROS) generation [58,59], we employed flow cytometry to determine ROS levels. Our findings corroborate the notion that elevated inflammatory marker expression is accompanied by augmented ROS expression, as observed in endothelial cells with P16Ink4a silencing. Subsequently, as increased ROS production is connected with endothelial cell dysfunction and leakage [59,60], we detected increased endothelial cell leakage upon silencing of P16Ink4a.

When considered collectively, P16Ink4a is believed to serve two distinct functions within endothelial cells, as proposed by the evolutionary “antagonistic pleiotropy” theory of senescence and aging. In accordance with the established functions of P16Ink4a as a negative regulator of the cell cycle and a marker of cellular senescence, our observations indicated an increase in proliferation of low levels of P16Ink4a, while a higher number of senescent cells was detected with rising levels of P16Ink4a expression. Furthermore, low levels of P16Ink4a have been observed to elicit an inflammatory response, ROS generation, and endothelial cell dysfunction. Conversely, high levels have been linked to the onset of senescence and the inhibition of angiogenesis. Additional research is required to elucidate the in vivo functions of P16Ink4a in pathological conditions related to endothelial dysfunction and age-related diseases with reduced angiogenesis. 

## Figures and Tables

**Table 1:** Primers used for quantitative RT-PCR.

Gene of Interest	Oligonucleotide Sequences
*p16ink4*	F: AGGGCCGTGTGCATGACGTG B: GCACCGGGCGGGAGAAGGTA
*Igf1R*	F: GTGGGGGCTCGTGTTTCT B: GATCTCCGTGCAGTTTTCCA
*Mmp2*	F: CAAGTTCCCCGGCGATGTC B: TTCTGGTCAAGGTCACCTGTC
*Mmp7*	F: CTGCCACTGTCCCAGGAAG B: GGGAGAGTTTTCCAGTCATGG
*Mmp9*	F: CCATGCACTGGGCTTAGATCA B: GGCCTTGGGTCAGGCTTAGA
*Mmp12*	F: GAGTCCAGCCACCAACATTAC B: GCGAAGTGGGTCAAAGACAG
*Timp1*	F: GCAACTCGGACCTGGTCATAA B: CGGCCCGTGATGAGAAACT
*Timp2*	F: TCAGAGCCAAAGCAGTGAGC B: GCCGTGTAGATAAACTCGATGTC
*Timp3*	F: CTTCTGCAACTCCGACATCGT B: GGGGCATCTTACTGAAGCCTC
*Timp4*	F: TGTGGCTGCCAAATCACCA B: TCATGCAGACATAGTGCTGGG
*E-selectin*	F: ATGCCTCGCGCTTTCTCTC B: GTAGTCCCGCTGACAGTATGC
*P-selectin*	F: CATCTGGTTCAGTGCTTTGATCT B: ACCCGTGAGTTATTCCATGAGT
*Anapc2*	F: TCCGATGACTGCGACTCTAGG B: CACTTCCACGAACCACTCCT
*Fos*	F: CGGGTTTCAACGCCGACTA B: TTGGCACTAGAGACGGACAGA
*Tnfα*	F: GTAGCCCACGTCGTAGCAAA B: ACAAGGTACAACCCATCGGC
*Mafk*	F: ATGACGACTAATCCCAAGCCC B: CGTAGCCTCTGTTCTTGAGTGT
*Mastl*	F: TCGGCAAGTGAGGAGAATGAA B: CACCACGGCTAATGGGCTT
*Terc*	F: GTGGGTTCTGGTCTTTTGTTCT B: CTGCAGGTCTGGACTTTCCT
*Tert*	F: TCAAGAGCAGTAGTCGCCAG B: TCTCGGGACAGGATAGCATCT
*Vegfa*	F: CTCACCAAAGCCAGCACATA B: AATGCTTTCTCCGCTCTGAA
*Casp3*	F: ATGGAGAACAACAAAACCTCAGT B: TTGCTCCCATGTATGGTCTTTAC
*Casp4*	F: ACAAACACCCTGACAAACCAC B: CACTGCGTTCAGCATTGTTAAA
*Ccl2*	F: AGCTGTAGTTTTTGTCACCAAGC B: GTGCTGAAGACCTTAGGGCA
*Ccl3*	F: TTCTCTGTACCATGACACTCTGC B: CGTGGAATCTTCCGGCTGTAG
*Ccl5*	F: GCTGCTTTGCCTACCTCTCC B: TCGAGTGACAAACACGACTGC
*Ccl9*	F: CCCTCTCCTTCCTCATTCTTACA B: AGTCTTGAAAGCCCATGTGAAA
*Ccl12*	F: ATTTCCACACTTCTATGCCTCCT B: ATCCAGTATGGTCCTGAAGATCA
*Cxcl10*	F: CCAAGTGCTGCCGTCATTTTC B: GGCTCGCAGGGATGATTTCAA
*Il-6*	F: CACTTCACAAGTCGGAGGCT R: TGCCATTGCACAACTCTTTTCT
*Il-8*	F: CAAGGCTGGTCCATGCTCC B: TGCTATCACTTCCTTTCTGTTGC
*Il-10*	F: GCTCTTACTGACTGGCATGAG B: CGCAGCTCTAGGAGCATGTG
*Il-18*	F: CAAAGTGCCAGTGAACCCCA B: TTCACAGAGAGGGTCACAGC
*NF-κB*	F: ATGGCAGACGATGATCCCTAC B: TGTTGACAGTGGTATTTCTGGTG
*Stat1*	F: TCACAGTGGTTCGAGCTTCAG B: GCAAACGAGACATCATAGGCA
*Stat2*	F: TCCTGCCAATGGACGTTCG B: GTCCCACTGGTTCAGTTGGT
*Tgfb1*	F: AGCTGGTGAAACGGAAGC G B: GCGAGCCTTAGTTTGGACAGG
*Tgfb2*	F: CCATCCCGCCCACTTTCTAC B: CATCAAAGCGGACGATTCTGA
*Tgfb3*	F: GGACTTCGGCCACATCAAGAA B: TAGGGGACGTGGGTCATCAC
*Ifna*	F: GACCTGCAAGGCTGTCTGAT B: AGACAGGGCTCTCCAGACTT
*Ifnar1*	F: AGCCACGGAGAGTCAATGG B: GCTCTGACACGAAACTGTGTTTT
*Ifnb*	F: CAGCTCCAAGAAAGGACGAAC B: GGCAGTGTAACTCTTCTGCAT
*Ifngr1*	F: CTGGCAGGATGATTCTGCTGG B: GCATACGACAGGGTTCAAGTTAT
*Gapdh*	F: AGGTCGGTGTGAACGGATTTG B: TGTAGACCATGTAGTTGAGGTCA
*β-actin*	F: CTTCCTCCCTGGAGAAGAGC B: ATGCCACAGGATTCCATACC
*Rplp0*	F: CACTGGTCTAGGACCCGAGAAG B: GGTGCCTCTGGAGATTTTCG

F: forward primer sequence; B: backward (reverse) primer sequence.

## Data Availability

All data are available on request. Sequencing data were uploaded to the GEO database (GSE281665).

## Supplementary Materials

The following supporting information can be downloaded at: https://www.mdpi.com/article/10.3390/cells13231929/s1, Figure S1: P16Ink4a overexpression reduces cell migration in wound healing assays in CD31+ cells.; Figure S2: P16Ink4a overexpression impairs migration in CD146+ cells.; Figure S3: Flow cytometry of Annexin-V-staining for detection of apoptotic cells.
