# Supplementary material for: A Dual Role of the Senescence Marker P16Ink4a in Liver Endothelial Cell Function
Source: Cells. 2024 Nov 21;13(23):1929. doi: 10.3390/cells13231929 (PMC11640762; doi:10.3390/cells13231929)
Supplement: Supplementary file 1 [file cells-13-01929-s001.zip › cells-3302213-supplementary.pdf]

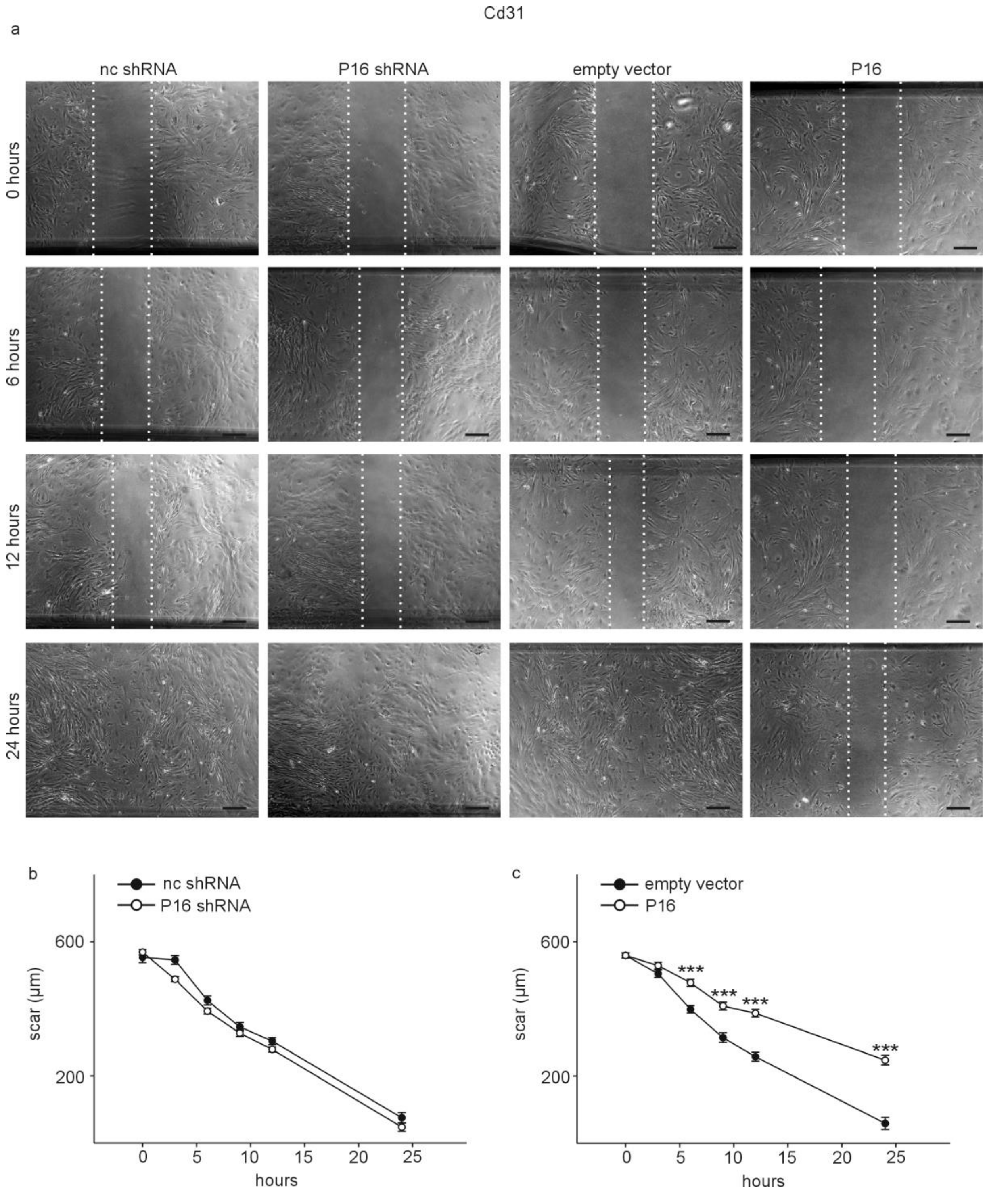

Supplementary Figure S1. P16Ink4a overexpression reduces cell migration in wound healing assays in CD31+ cells. (**a**) Representative photomicrographs of Cd31+ cells at 0, 6, 12, and 24 hours after scratch. Cells transduced with non-coding small hairpin plasmids (nc shRNA) served as control for P16Ink4a silencing plasmid (P16 shRNA) transduced cells. Empty vector controls were compared to cells with overexpression of P16Ink4a (P16). The scar width was determined at 0, 3, 6, 9, 12, and 24 hours after the scratch for cells following silencing (**b**) or overexpression (**c**) of P16Ink4a compared to the respective controls. Dotted lines mark the border of the wound. The data are presented as the mean ± SEM (*n*=6, each). ***$p<0.001$.

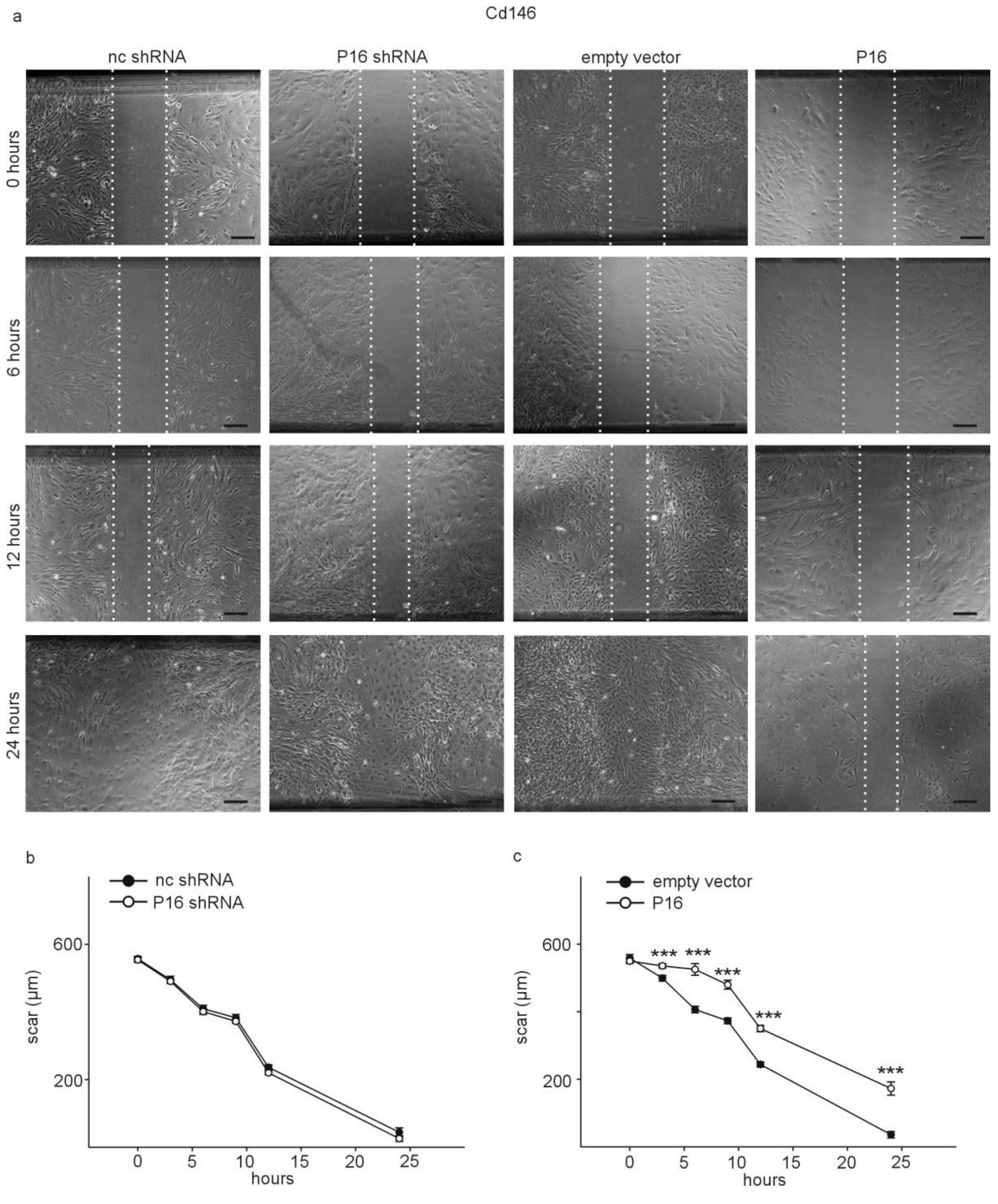

Supplementary Figure S2. P16Ink4a overexpression impairs migration in CD146+ cells. (**a**) Representative photomicrographs of Cd146+ cells at 0, 6, 12, and 24 hours after scratch. Cells transduced with non-coding small hairpin plasmids (nc shRNA) served as control for P16Ink4a silencing plasmid (P16 shRNA) transduced cells. Empty vector controls were compared to cells with overexpression of P16Ink4a (P16). The scar width was determined at 0, 3, 6, 9, 12, and 24 hours after the scratch for cells following silencing (**b**) or overexpression (**c**) of P16Ink4a compared to the respective controls. Dotted lines mark the border of the wound. The data are presented as the mean ± SEM (*n*=6, each). ***$p<0.001$.

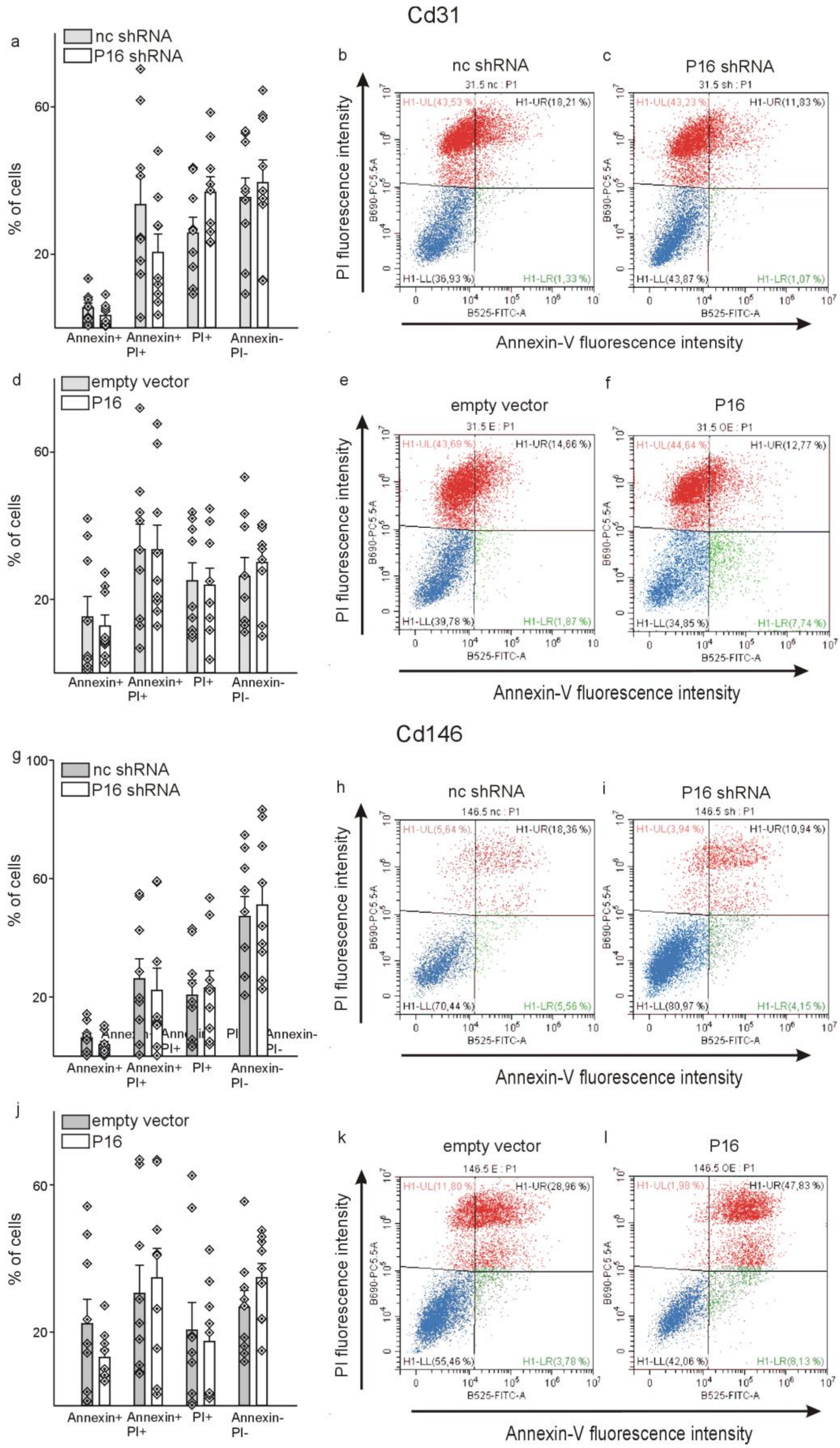

Cd31
a
nc shRNA
P16 shRNA
% of cells
Annexin+
Annexin+ PI+
PI+
Annexin- PI-
b
nc shRNA
c
P16 shRNA
PI fluorescence intensity
Annexin-V fluorescence intensity
H1-UL(43,53 %)
H1-UR(18,21 %)
H1-LL(36,93 %)
H1-LR(1,33 %)
H1-UL(43,23 %)
H1-UR(11,83 %)
H1-LL(43,87 %)
H1-LR(1,07 %)
d
empty vector
P16
e
empty vector
f
P16
H1-UL(43,69 %)
H1-UR(14,66 %)
H1-LL(39,78 %)
H1-LR(1,87 %)
H1-UL(44,64 %)
H1-UR(12,77 %)
H1-LL(34,85 %)
H1-LR(7,74 %)
Cd146
g
nc shRNA
P16 shRNA
h
nc shRNA
i
P16 shRNA
H1-UL(5,64 %)
H1-UR(18,36 %)
H1-LL(70,44 %)
H1-LR(5,56 %)
H1-UL(3,94 %)
H1-UR(10,94 %)
H1-LL(80,97 %)
H1-LR(4,15 %)
j
empty vector
P16
k
empty vector
l
P16
H1-UL(11,80 %)
H1-UR(28,96 %)
H1-LL(55,46 %)
H1-LR(3,78 %)
H1-UL(1,98 %)
H1-UR(47,83 %)
H1-LL(42,06 %)
H1-LR(8,13 %)
B690-PC5.5-A
B525-FITC-A

Supplementary Figure S3. Flow cytometry of Annexin-V- and propidium iodide (PI) staining for detection of early apoptotic (Annexin+), late apoptotic and necrotic (Annexin+/PI+; PI+), and live (Annexin-/PI-) cells. Cd31+ (**a**-**f**) and Cd146+ (**g**-**l**) cells were incubated with Annexin-V/PI one week after transduction with P16Ink4a silencing or overexpression constructs and the respective controls and the cells analysed by flow cytometry. Annexin-V labels apoptotic cells while PI is used to exclude necrotic cells. (**a**) Percentage of cells with the different Annexin/PI labelling in Cd31+ samples following silencing of P16Ink4a (P16 shRNA) and the respective control (nc shRNA). Representative examples for control (**b**) and P16Ink4a silencing (**c**). (**d**) Percentage of cells with the different Annexin/PI labelling in Cd31+ samples following overexpression of P16Ink4a (P16) and the respective control (empty vector). Representative examples for control (**e**) and P16Ink4a overexpression (**f**). (**g**) Percentage of cells with the different Annexin/PI labelling in Cd146+ samples following silencing of P16Ink4a and the respective control. Representative examples for control (**h**) and P16Ink4a silencing (**i**) in Cd146+ cells. (**j**) Percentage of cells with the different Annexin/PI labelling in Cd146+ samples following overexpression of P16Ink4a (P16) and the respective control (empty vector). Representative examples for control (**k**) and P16Ink4a overexpression (**l**). The data are presented as the mean ± SEM (*n*=9, each). Symbols indicate individual values.
